# Evaluating a shared care pathway intervention for people receiving chemotherapy to reduce post-treatment unplanned hospital presentations: a randomised controlled trial

**DOI:** 10.1007/s00520-023-08261-w

**Published:** 2024-01-03

**Authors:** Judith Fethney, Bora Kim, Chantale Boustany, Heather McKenzie, Lillian Hayes, Keith Cox, Judy M. Simpson, Lisa G. Horvath, Janette L. Vardy, Jodi McLeod, Simon Willcock, Natalie Cook, Louise Acret, Kate White

**Affiliations:** 1 Susan Wakil School of Nursing and Midwifery, The University of Sydney, Sydney, Australia; 2 Cancer Care Research Unit, Sydney Local Health District, The University of Sydney, Sydney, Australia; 3 The Daffodil Centre, The University of Sydney, a joint venture with Cancer Council NSW, Sydney, Australia; 4 Chris O’Brien Lifehouse, Sydney, Australia; 5 Sydney School of Public Health, The University of Sydney, Sydney, Australia; 6 Faculty of Medicine and Health, The University of Sydney, Sydney, Australia; 7 Sydney Local Health District, Sydney, Australia; 8 Sydney District Nursing, Sydney Local Health District, Sydney, Australia; 9 MQ Health, Macquarie University Hospital, Primary Care, Sydney, Australia; 10 Healthdirect, Sydney, Australia

**Keywords:** Randomised controlled trial, Systemic chemotherapy, Unplanned presentations, Unplanned admissions, Community nursing intervention, Quality of life

## Abstract

**Purpose:**

The aim of this randomised controlled trial (RCT) was to explore whether a community nursing intervention for outpatients receiving systemic therapy reduced unplanned hospital presentations and improved physical and psychosocial health outcomes over the first three cycles of treatment compared to a control group receiving standard care.

**Methods:**

The number of and reasons for unplanned presentations were obtained for 170 intervention and 176 control group adult patients with solid tumours starting outpatient chemotherapy. Poisson regression was used to compare the number of presentations between the intervention and control groups. Patients self-completed the Hospital Anxiety and Depression Scale, the Cancer Behavior Inventory and the European Organization for Research and Treatment of Cancer Quality of Life Questionnaire core 30 (EORTC QLQ-C30) at the start of the first four cycles. Linear regression techniques were used to compare quality of life outcomes.

**Results:**

The reduction in unplanned presentations in the intervention group relative to the control group was 12% (95% CI, − 25%, 37%; *P* = 0.48). At the start of cycle 4, there was no difference in anxiety (difference = 0.47 (95% CI, − 0.28, 1.22; *P* = 0.22)), depression (difference = 0.57 (95% CI, − 0.18, 1.31; *P* = 0.13)) or EORTC QLQ-C30 summary score (difference = 0.16 (95% CI, − 2.67, 3.00; *P* = 0.91)). Scores for self-efficacy as measured by the Cancer Behavior Inventory were higher in the intervention group (difference = 4.3 (95% CI, 0.7, 7.9; *P* = 0.02)).

**Conclusion:**

This RCT did not demonstrate a benefit in reducing unplanned presentations to hospital. The trial identified improved cancer-based self-efficacy in patients receiving the intervention.

**Trial registration:**

Registered at Australian and New Zealand Clinical Trials Registry: ACTRN12614001113640, registered 21/10/2014.

**Supplementary Information:**

The online version contains supplementary material available at 10.1007/s00520-023-08261-w.

## Introduction and background

Reducing unplanned presentations to hospital, including those leading to admission, has the potential to improve the quality of cancer care while reducing health costs. This dual advantage means that reducing admissions is an area of policy interest in a number of countries [1]. 

In Australia, reducing avoidable hospital presentations for people with chronic conditions was a focus of the ‘Keeping Australians out of Hospital’ initiative. This initiative funded projects aimed at decreasing avoidable hospital presentations, helping community-based health services put research findings into practice and helping people better manage their chronic and complex health conditions [2]. In a 5-year (2020–2025) National Health Agreement (NHA), the Commonwealth and State governments agreed to decrease demand for public hospital services through better coordinated care, particularly for patients with complex and chronic diseases [3].

Outpatients receiving systemic anti-cancer therapy (SACT) are at risk of making unplanned presentations to hospital for management of systemic chemotherapy side effects. A retrospective study of the magnitude and nature of unplanned presentations to both the Chemotherapy Day Therapy Unit and emergency department at a large Sydney, Australia, teaching hospital demonstrated that over a 12-month period (i) 45% of systemic chemotherapy outpatients made unplanned presentations to hospital within 6 months of receiving treatment, primarily due to its side effects, and (ii) 87% of those who made unplanned presentations in treatment cycle 1, 2 or 3 required admission to hospital, with a median length of stay of 6 days [4]. The three primary reasons for unplanned presentations were nausea/vomiting (45%), pain (27%) and fever/febrile neutropenia (23%), with most presentations occurring in the first 2 weeks of a treatment cycle.

In Australia, 247,939 patients were treated with systemic chemotherapy in 2016, an increase of 25% from 2012 [5]. The majority received these therapies as outpatients. In addition to managing cancer treatment side effects, patients also have to manage the psychosocial consequences of a cancer diagnosis [6, 7]. Quality of life, anxiety, depression and self-efficacy have been acknowledged as clinically important outcomes of cancer care, with recommendations to include these measurements in cancer clinical trials to inform patient-centred care [8].

Studies designed to reduce unplanned hospital utilisation in cancer care — a nurse-led symptom management clinic in the USA [9] and a nurse-delivered telephone supportive care service in Australia [10, 11] — did not show a significant reduction in unplanned hospital utilisation, predominantly due to sample size issues and participants reporting that local health services were sufficient, highlighting the need for an adequately powered study offering face-to-face, in-home consultations providing information and support that is not readily available elsewhere.

Recently, there has been widespread recognition of the advanced skills and extended scope of practice of community nurses (CNs). In NSW, cancer patients are a major diagnostic group receiving CN services [12]. A UK randomised controlled trial (RCT) demonstrated that timely involvement of community-based specialist cancer nurses significantly reduced health service utilisation, including unplanned hospital presentations and symptom burden for patients receiving chemotherapy, particularly during the first two treatment cycles [13]. Intervention duration, the timing of visits and phone calls, early referral and non-pharmacological support were identified as key to the effectiveness of the intervention.

This paper reports the results of a study in which patients receiving SACT were visited at home by CNs on days three and five of their first three treatment cycles. Evaluating a Shared Care Pathway Intervention to reduce systemic chemotherapy outpatients’ unplanned presentations to hospital (ESCAPI) is an RCT in which participants were randomised to receive either the CN visits or standard care. The aims of the study were to determine whether the intervention:

1. Reduced the number of unplanned hospital presentations by systemic chemotherapy outpatients; and
2. Improved systemic chemotherapy outpatients’ physical and psychosocial health outcomes.

## Methods

### Design and setting

This prospective RCT took place between August 2015 and January 2019 in two hospitals with specialist cancer centres in metropolitan Sydney NSW, Australia. The study had ethics approvals from the relevant hospital research integrity committee (RPAH Zone protocol X13-0101). All patients who agreed to participate gave fully informed written consent. We followed the CONSORT statement guidelines [14].

The randomisation sequence was generated by a biostatistician involved in the study (JMS) and stratified by site (cancer centre), using 1:1 allocation and random permuted blocks of size 6 or 8 (http://www.randomization.com). Opaque, sequentially numbered envelopes containing the group assignment as per the randomisation sequence were prepared by the project manager (CB). All researchers were blinded to group allocation, but patients and the project manager could not be blinded.

The CNs were employed at Community Health Centres within two health districts in metropolitan Sydney. Participating CNs completed an education program developed in collaboration with the Cancer Institute NSW, which used self-guided online modules. The education program included cancer and treatment updates, clinical protocols, managing side effects, clinical assessment, communication skills, patient education, treatment and referral pathways inclusive of triage category. In addition, CNs were provided with contact details for relevant cancer centre staff, if required. Researchers held regular meetings with participating CN centre staff to provide updates and discuss issues arising across all centres.

### Sample

Individuals diagnosed with cancer who were about to commence a systemic chemotherapy regimen at the two hospitals were invited to participate. Inclusion criteria were as follows: solid cancer tumour, over 18 years of age, starting the first cycle as an outpatient, fully aware of cancer diagnosis and living within the relevant health district. Exclusion criteria were as follows: being unable to give informed consent, being treated for haematological malignancy, receiving oral chemotherapy, receiving concurrent radiotherapy and not understanding written or spoken English. ESCAPI was communicated to GPs (general practitioners) in the health district via the Australian government Primary Care Networks Newsletter. Participants were asked, prior to randomisation, whether they consented to their GP being involved in the clinical pathway. If the participant consented, the GP received copies of the assessment made by the CN during the home visit, and CNs were also able to contact the GP if required provided the participant had been randomised to the intervention group.

### Standard care

Currently in Australia, there are no national guidelines for standardised care for systemic chemotherapy outpatients. Standard care at the two participating hospitals involved the provision of written material, an education session with a cancer nurse before treatment started, and advice to call the treatment unit within the hospital if they were unwell.

### Intervention

**Fig. 1:**
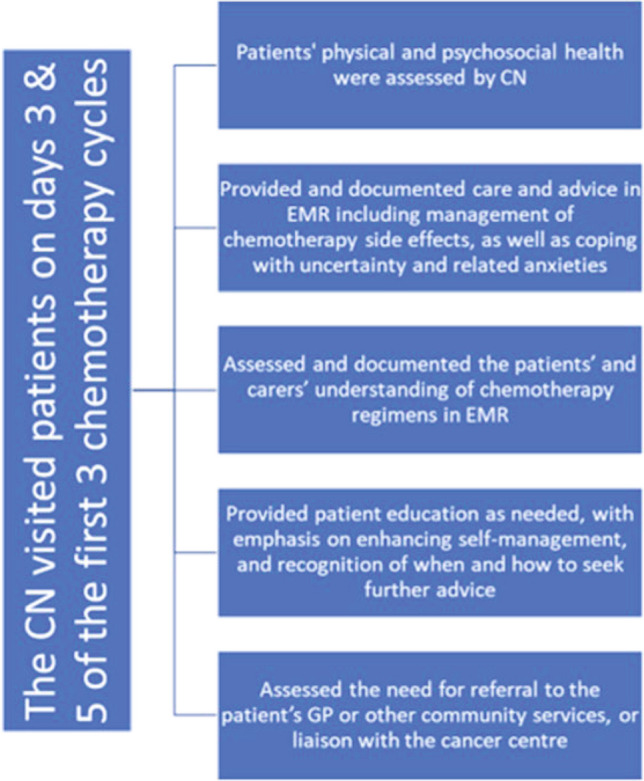
Intervention flowchart

The intervention involved CNs visiting patients in their homes on days three and five of the first three treatment cycles (see Fig. 1). CNs used the Chemotherapy Symptom Assessment Scale (C-SAS) [15] to assess patients, which was faxed through to the relevant cancer centre and patient’s GP, if participating. Care was therefore shared between CNs, cancer centre staff, participating GPs and any other services/health professionals referred to by the CN. Ten percent of randomly selected visits were audiotaped to monitor and maintain intervention fidelity.

### Outcome measures

The primary outcome was the number of all-cause unplanned presentations made to either the patient’s cancer centre or a hospital emergency department from day 1 of cycle 1 to day 1 of cycle 4 (thereby covering the first three cycles). Secondary outcomes included the following patient self-reported outcomes (PROs): the Hospital Anxiety and Depression Scale (HADS), the Cancer Behavior Inventory — Brief form (CBI-B), and the European Organization for Research and Treatment of Cancer Quality of Life Questionnaire (EORTC QLQ-C30). These instruments are widely used in oncology research and have satisfactory validity and reliability (see Supplement, Resource 1).

The HADS measures both anxiety and depression [16]. It is a 14-item questionnaire with responses ranging from 0 (best) to 3 (worst). There are 7 items on each scale; scores for anxiety and depression range from 0 to 21, while the total HADS score ranges from 0 to 42. Higher scores indicate greater anxiety, depression or distress.

The CBI-B is a single-score measure of coping self-efficacy [17]. We used the 14-item version. Responses range from 1 (not at all confident) to 9 (totally confident). Summing the scores for the 14 items gives the total score (out of 126). Higher scores indicate greater coping efficacy.

The EORTC QLQ-C30 is a cancer-specific 30-item questionnaire measuring physical, role, emotional, cognitive and social function, cancer symptoms and global quality of life (QoL) [18]. For the five function scales and the QoL scale, higher scores indicate better health. For the nine symptom scales, higher scores indicate greater symptom burden. All scale scores are transformed to range from 0 to 100. We used the QLQ-C30 summary score [19], which is calculated as the mean of the combined 13 QLQ-C30 scale scores (excluding financial impact and QoL); all included symptom scale scores are reversed so that higher scores represent better outcomes. The use of EORTC QLQ-C30 summary score avoids potential problems with multiple testing of 15 outcomes [20].

### Data collection

Demographic data and pre-treatment baseline measures for all participants were collected before randomisation. Primary outcome data was obtained from the Electronic Medical Record (EMR). PROs were collected on electronic tablets on day 1 of cycles 1 to 4 using Survey Monkey. Patients who made an unplanned presentation to a hospital outside the local health district provided patient-reported data relating to the presentation. As previous research has found a considerable number of patients making unplanned presentations are also admitted [4], admissions information was also obtained from the EMR as was comorbidity prevalence in this patient cohort. Comorbidities were recorded in the EMR as free text by the clinical staff. Patients who ceased all treatment or had treatment modified to receive immunotherapy- or oral chemotherapy-only, concurrent chemotherapy with radiotherapy or radiotherapy-only regime no longer received the intervention as their side effects profile did not conform to traditional chemotherapy side effects, and the CNs were not trained to manage these. Patients with treatment modified to include radiotherapy make daily visits to the cancer centre, which would have made it difficult to identify the effect of the intervention from more regular cancer centre contact.

### Sample size

We used data from the McKenzie et al. study [4] in which 363 chemotherapy outpatients had a mean of 0.56 (SD 0.96) unplanned presentations. Informed by the reduction in health service utilisation reported in Molassiotis et al. [13], we determined that to detect a 35% reduction in number of presentations by the start of cycle 4, with 5% two-sided significance and 80% power, we needed a total sample size of 326 (Poisson regression procedure in PASS), with sample size increased by 20% to 408 to allow for non-compliance and loss to follow-up.

### Data analysis

All analyses were by intention-to-treat and blinded to treatment group. Data were analysed using SPSS V26 and Stata 14. No adjustment was made for multiple comparisons because all analyses were pre-specified unless noted to be post hoc.

#### Primary outcome

The total number of unplanned presentations was aggregated across cycles 1, 2 and 3. The primary analysis used Poisson regression (due to dispersion of the data) adjusted for site, with the natural log of time at risk of making an unplanned presentation included as the offset variable. In the secondary analysis, we also adjusted for age, informal carer (family or friend), cancer type and cancer stage. Sex was not included as there was a strong relationship between cancer type and sex due to the large numbers of female-only cancers.

#### Time at risk

The time at risk of making an unplanned presentation was calculated as the aggregated time from the start of cycle 1 to the start of cycle 4, or whichever was the final cycle for patients who did not start all 4 cycles. For patients who died before the start of cycle 4, the date of death was used as the end of study. If a patient had a delay to the start of a cycle of ≤ 7 days, no adjustment was made to time at risk. If the delay was > 7 days, then:

1. If the delay was due to chemotherapy-induced toxicities or disease progression, the time at risk included the delay, as the patient was considered to be at risk of making an unplanned presentation over that duration.
2. If the delay was not disease- or treatment-related (e.g. due to social reasons or planned surgery), the expected duration of the cycle based on the chemotherapy protocol was used rather than actual duration, as the patient was considered not to be at risk of an unplanned presentation beyond the expected duration of the cycle.

Time at risk excluded days spent in hospital during the particular chemotherapy cycle.

#### Secondary outcomes

Secondary outcomes were compared at the end of cycle 3, as per trial protocol and statistical analysis plan. Missing values were imputed using values at all other cycles. Because all the secondary outcomes were patient-reported, patients who died before the end of cycle 3 were excluded from the analysis; their results could not be validly imputed because they were not missing at random. Each primary analysis was adjusted for site and baseline value of the outcome using multiple linear regression. As the following covariates have been reported to be associated with unplanned presentations or psychosocial responses to cancer, secondary analyses were also adjusted for age [21], carer [22–25], cancer type and cancer stage [26].

#### Missing data

For each treatment cycle started, there was no missing data for the number of and reasons for unplanned presentations, as it was ascertained from the EMR. For HADS, if any item was missing in a subscale (anxiety or depression), the patient’s mean of all non-missing items was substituted, regardless of how many were missing [27]. If all items in a subscale were missing, the subscale value was imputed by multiple imputations using chained equations in Stata, with predictive mean matching with 10 nearest neighbours for the continuous outcome and 40 imputations [28]. The same approach was used for the single-scale CBI-B using 35 imputations. For the EORTC summary score, the patient’s mean of all non-missing items was substituted provided at least half the scale items were present [18]. If more than half were missing, we used multiple imputations with 35 imputations, as for the CBI-B. Among the covariates, there was no missing data for site, age and cancer type; missing data for carer (5%) and cancer stage (9%) were imputed during the multiple imputation.

## Results

**Fig. 2:**
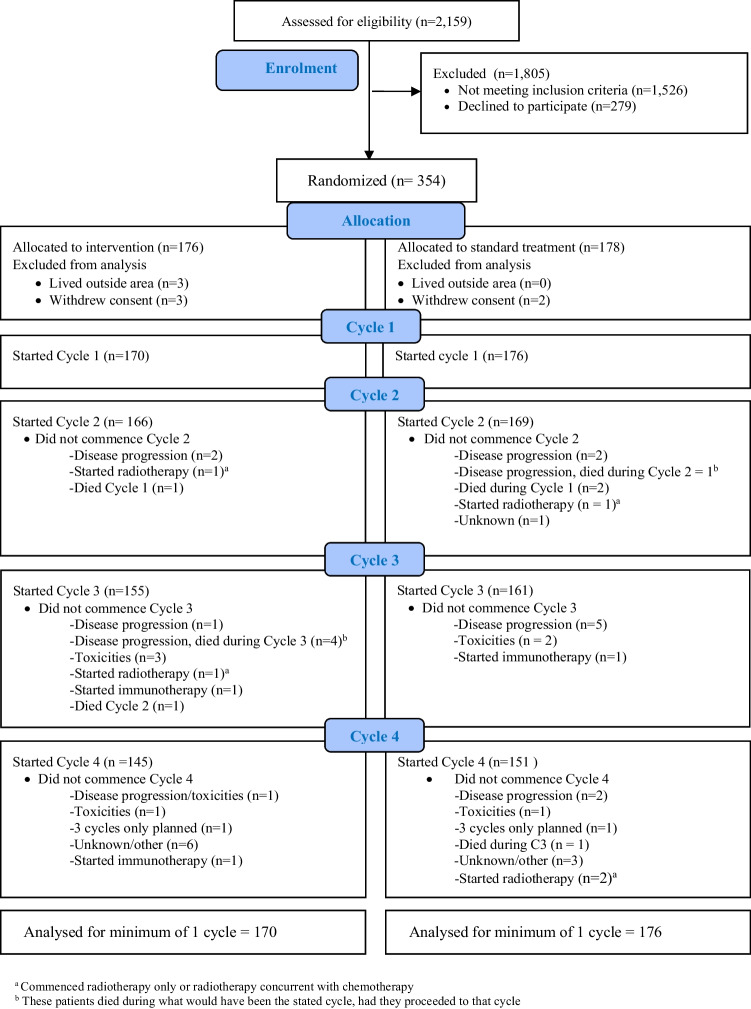
Participant flowchart

A total of 354 patients were randomised, with primary outcome assessment available for 346 patients (98%) (Fig. 2). Adherence information is provided in the Supplement, Resource 2. In both the intervention (*n* = 170) and control group (*n* = 176), the majority were female (67%, 62%), married/de facto (70%, 67%), had an informal carer (83%, 86%) and were on a 21-day cycle treatment protocol (66%, 66%); curative in intent (42%, 42%) and the most common diagnosis was breast cancer (33%, 23%) (Table 1). There were no adverse events due to the intervention. Seventy-five CNs completed the education modules and delivered the intervention. Consent to involve the GP was granted by 90% of the total sample.

**Table 1:** Characteristics of participants at baseline by treatment group

	Intervention *N* = 170 *n* (%)	Control *N* = 176 *n* (%)
Gender		
Male	56 (32.9)	67 (38.1)
Female	114 (67.1)	109 (61.9)
Age Mean (SD)	59.3 (12.7)	59.4 (13.6)
(range)	24 to 87	30 to 85
Marital status		
Never married	28 (16.5)	26 (14.8)
Married/de facto	119 (70.0)	117 (66.5)
Separated/divorced	15 (8.8)	15 (8.5)
Widowed	6 (3.5)	14 (8.0)
Missing	2 (1.2)	4 (2.3)
Informal carer		
Yes	141 (82.9)	151 (85.8)
No	21 (12.4)	17 (9.7)
Missing	8 (4.7)	8 (4.5)
Type of cancer		
Breast	56 (32.9)	41 (23.3)
Colorectal	20 (11.8)	30 (17.0)
Genito-urinary	18 (10.6)	15 (8.5)
Gynaecological	33 (19.4)	24 (13.6)
Lung	21 (12.4)	20 (11.4)
Upper gastrointestinal	19 (11.2)	31 (17.6)
Other	3^a^ (1.8)	15^b^ (8.5)
Cancer stage		
I	7 (4.1)	6 (3.4)
II	25 (14.7)	30 (17.0)
III	46 (27.1)	31 (17.6)
IV	79 (46.5)	90 (51.1)
Missing	13 (7.6)	19 (10.8)
Cycle duration based on chemotherapy protocol		
7 days	5 (2.9)	4 (2.3)
14 days	40 (23.5)	38 (21.6)
21 days	112 (65.9)	116 (65.9)
28 days	13 (7.6)	18 (10.2)
Intent of treatment		
Curative	72 (42.4)	73 (41.5)
Palliative	59 (34.7)	65 (36.9)
Not specified	39 (22.9)	38 (21.6)
Number of co-morbidities		
Mean (SD)	1.4 (1.7)	1.7 (1.6)
Min-Max	0–9	0–7
Median (IQR)	1.0 (0.0–2.0)	1.0 (0.0–3.0)

^a^1 × Mesothelioma, 1 × Head and Neck, 1 × Sarcoma

^b^4 × Mesothelioma, 3 × Peritoneal, 3 × Head and Neck, 2 × Metastatic adenocarcinoma, 1 × Sarcoma, 1 × Brain cancer, 1 × Unknown primary

In the intervention group, 106 (62%) patients and 126 (72%) patients in the control group reported comorbidities. The most frequently reported comorbidities for the intervention vs control group were hypertension (25%, 31%), hypercholesterolaemia (9%, 7%), diabetes (9%, 11%), coronary heart disease (5%, 7%) and depression (7%, 5%).

Cycle delivery and completion details are reported in Table 2. In both groups, most participants started all 4 cycles (85.0%, 86.0%). Ten patients died during the study, 6 in the intervention group and 4 in the control group. In both groups, most participants completed their cycles with no treatment delay or delays < 7 days (85%, 88%). Additional information about delays in treatment is in the Supplement, Resource 3.

**Table 2:** Cycle delivery, completion and delays of participants by treatment group

	Intervention *N* = 170 *n* (%)	Control *N* = 176 *n* (%)
Cycles commenced		
Cycle 1 only	4 (2.4)	7 (4.0)
Cycle 1 & 2	11 (6.5)	8 (4.5)
Cycle 1, 2 & 3	10 (5.9)	10 (5.7)
Cycle 1, 2, 3 & 4	145 (85.3)	151 (85.8)
Delay to treatment > 7 days		
No	144 (84.7)	155 (88.1)
Yes	26 (15.3)	21 (11.9)
Deceased	6 (3.5)	4 (2.3)
Mean (SD) time at risk (days)	57.3 (16.4)	57.9 (16.2)
Range	8 to 112	3 to 97
Median (IQR)	63 (42–63)	63 (47–64)

### Primary outcome: Unplanned presentations

**Fig. 3:**
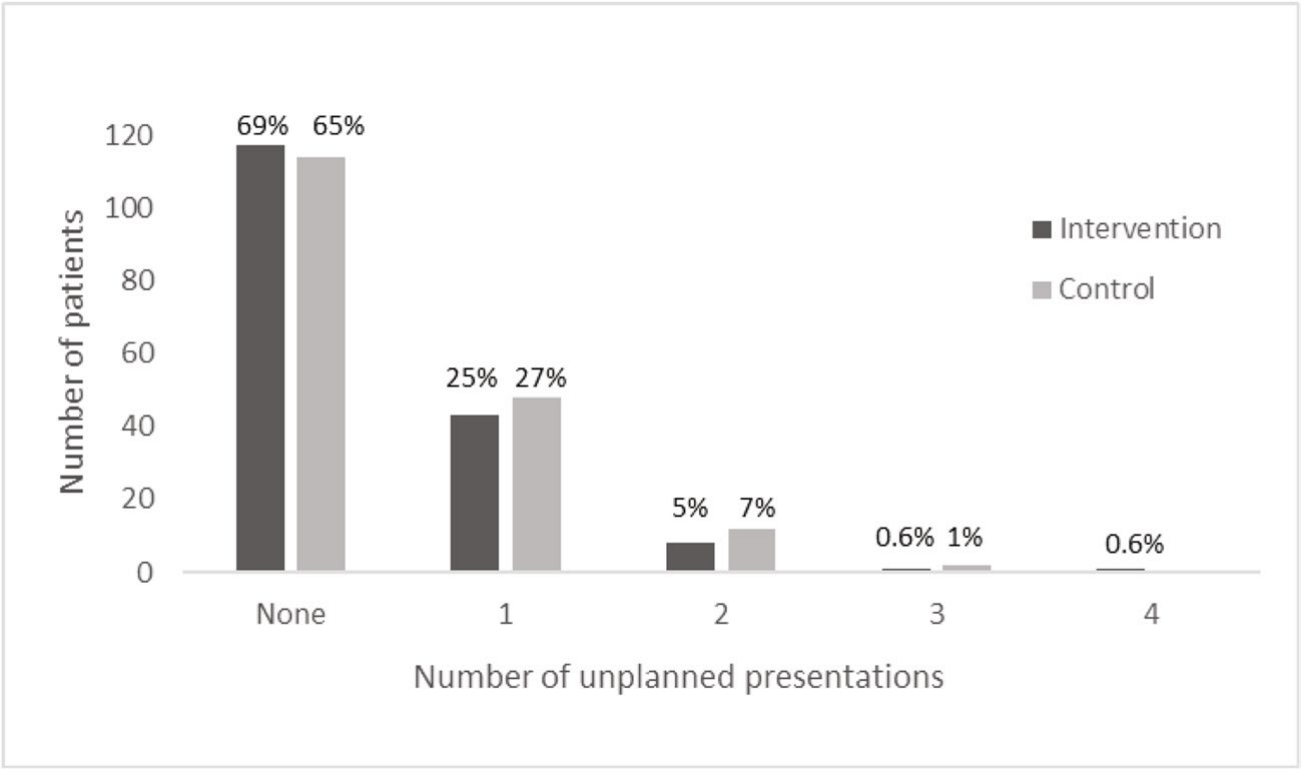
Unplanned presentations to hospital by treatment group

One or more unplanned presentation was made by 53 patients (31%) in the intervention group and 62 patients (35%) in the control group, resulting in a total of 66 (mean 0.39) unplanned presentations in the intervention group and 78 ( mean 0.44) in the control group (Fig. 3). Using Poisson regression to adjust for time at risk and site, the relative reduction in the number of unplanned presentations in the intervention compared to the control group was 12% (95% CI, − 25%, 37%; *P* = 0.48) and in the secondary analysis 10% (95% CI, − 28%, 36%; *P* = 0.56), after also adjusting for age, carer, cancer type and cancer stage. In both groups, most participants who made an unplanned presentation did so within the first 7 days of a chemotherapy cycle, mainly due to chemotherapy side effects. Most presentations (51%) were made during cycle 1 (Table 3). Only one patient presented to a hospital outside the local health district.

**Table 3:** Characteristics of unplanned presentations by treatment group

	Intervention *N* = 66/170 *n* (%)	Control *N* = 78/176 *n* (%)
Days from start of chemotherapy cycle to unplanned presentation		
1−7 days	29 (43.9)	31 (39.7)
8−14 days	25 (37.9)	26 (33.3)
15−21 days	9 (13.6)	18 (23.1)
22−28 days	2 (3.0)	1 (1.3)
29−43 days	1 (1.5)	2 (2.6)
Presented during cycle		
1	36 (54.6)	38 (48.7)
2	17 (25.8)	23 (29.5)
3	13 (19.7)	17 (21.8)
Reason for presentation		
Chemotherapy side effects	38 (57.6)	43 (55.1)
Disease progression	13 (19.7)	16 (20.5)
Unrelated to chemotherapy or disease	5 (7.6)	6 (7.7)
Combination of reasons	10 (15.2)	13 (16.7)
Symptom at presentation^a^		
Fever without febrile neutropenia	14 (21.2)	13 (16.7)
Febrile neutropenia	11 (16.7)	12 (15.4)
Nausea and vomiting	11 (16.7)	19 (24.4)
Abdominal pain	10 (15.2)	11 (14.1)
Diarrhoea	5 (7.6)	9 (11.5)
Constipation	6 (9.1)	5 (6.4)
Shortness of breath	6 (9.1)	7 (9.0)
Lethargy/Fatigue	6 (9.1)	6 (7.7)
Cough/sore throat	5 (7.7)	12 (15.6)
Confusion	4 (6.1)	2 (2.6)
Dehydration	2 (3.0)	5 (6.4)
Anxiety	3 (4.5)	4 (5.1)
Chest pain	5 (7.6)	4 (5.1)
Headache	3 (4.5)	4 (5.1)
Collapse	4 (6.1)	1 (1.3)
Number of symptoms (Mean, SD)	2.2 (1.3)	2.3 (1.42)
Range	1–7	1–8

^a^Numbers add up to more than 66 and 78 and percentages add up to more than 100 as some patients presented with multiple symptoms

In total, 62 reasons for making an unplanned presentation were identified, with patients in both groups presenting with an average of 2 symptoms. The presenting symptoms, aggregated across all 3 cycles, that were reported for at least 5% in either group, are shown in Table 3. In both the intervention and control group, most presentations for fever occurred during the first 7 days following treatment (64%, 31%), for febrile neutropenia within 8–14 days (73%, 58%) and for nausea and vomiting within the first 7 days (64%, 53%).

### Unplanned admissions

There were 37 (mean 0.22) and 49 (mean 0.27) unplanned admissions to hospital. In a post hoc analysis using negative binomial regression to allow for time at risk, the reduction in admissions following an unplanned presentation in the intervention relative to control group was 17% (95% CI, − 37%, 50%, *P* = 0.46). Symptoms that were diagnosed on admission in ≥ 5% of either group are reported in Table 4, aggregated across all cycles.

**Table 4:** Characteristics of all unplanned hospital admissions by treatment group

	Intervention *N* = 37/170 *n* (%)	Control *N* = 49/176 *n* (%)
Reason for admission^a^		
Febrile neutropenia	14 (37.8)	12 (24.5)
Respiratory infection	8 (21.6)	7 (14.3)
Fever without febrile neutropenia	5 (13.5)	13 (26.5)
Abdominal pain	6 (16.2)	6 (12.2)
Nausea and vomiting	6 (16.2)	11 (22.4)
Diarrhoea	5 (13.5)	7 (14.3)
Constipation	5 (13.5)	6 (12.2)
Shortness of breath	5 (13.5)	2 (4.1)
Anaemia	5 (13.5)	3 (6.1)
Electrolyte imbalance	3 (8.1)	6 (12.2)
Dehydration	2 (5.4)	4 (8.2)
Chest pain	2 (5.4)	4 (8.2)
Collapse	2 (5.4)	0 (0.0)
Back pain	2 (5.4)	0 (0.0)
Decreased consciousness	2 (5.2)	1 (2.0)
Confusion	2 (5.4)	1 (2.0)
Bleeding	2 (5.4)	1 (2.0)
Thrombocytopenia	2 (5.4)	0 (0.0)
Tachycardia	2 (5.4)	0 (0.0)
High creatinine	2 (5.4)	0 (0.0)
Lethargy/Fatigue	1 (2.7)	3 (6.1)
General weakness	1 (2.7)	3 (6.1)
Urinary tract infection	0 (0.0)	5 (10.2)
Pleural effusions	0 (0.0)	4 (8.2)
Number of symptoms (Mean, SD)	3.1 (2.3)	2.9 (1.5)
Min-Max	1–11	1–8
Total length of stay days (Median, IQR)	3.0 (2.0–9.0)	3.0 (2.0–7.0)

^a^Numbers add up to more than 36 and 50 and percentages add up to more than 100 as some patients admitted with multiple symptoms

### Secondary outcomes

Results of the primary analysis of the PROs for the 336 patients who survived to the start of cycle 4 are shown in Table 5. For the HADS, after adjusting for site and baseline score, the final anxiety score was reduced by slightly more in the intervention than the control group by 0.47 (95% CI, − 0.28 to 1.22; *P* = 0.22). Depression scores were increased by slightly less in the intervention than the control group, a difference of 0.57 (95% CI, − 0.18 to 1.31; *P* = 0.13). The total HADS score showed that distress scores were decreased by somewhat more in the intervention than the control group, a difference of 1.05 (95% CI, − 0.25 to 2.35; *P* = 0.12). Results for all HADS scales were similar in the secondary analysis, which also showed that final anxiety scores were higher by an average of 1.45 (95% CI, 0.32 to 2.59; *P* = 0.01) among patients with a carer than those without.

**Table 5:** Comparison of secondary outcomes between groups

	Intervention (*N*=164)		Control (*N*=172)			Difference^a^ at end Intervention – Control		*P*-value
	Baseline	End of cycle 3	Change	Baseline	End of cycle 3	Change		
HADS								
Anxiety	7.24	5.54	−1.70	6.68	5.67	−1.00	−0.47 (−1.22, 0.28)	0.22
Depression	4.31	4.81	0.50	4.39	5.42	1.03	−0.57 (−1.31, 0.18)	0.13
Total (distress)	11.55	10.35	−1.20	11.07	11.10	0.03	−1.05 (−2.35, 0.25)	0.12
CBI-B	97.2	102.5	5.3	99.3	99.5	0.2	4.3 (0.7, 7.9)	0.02
EORTC summary score	76.21	72.76	−3.46	76.03	72.83	−3.20	−0.16 (−3.00, 2.67)	0.91

^a^Difference between groups at end of Cycle 3 is adjusted for site and baseline value

For CBI-B, where higher scores indicate greater coping efficacy, the increase was greater in the intervention group by 4.3 (95% CI, 0.7 to 7.9; *P* = 0.02). There was no evidence of any effect of the intervention on the EORTC QLQ-C30 summary score, for which higher scores indicate better quality of life. Scores were reduced by only 0.16 more in the intervention group (95% CI, − 2.67, 3.00; *P* = 0.9) (see Supplement, Resource 4 for all 15 subscale results). Results were again similar in the secondary analyses of both CBI-B and QLQ-C30 summary scores. For CBI-B, patients without a carer had greater coping self-efficacy than those with a carer by an average of 6.4 (95% CI, 1.0 to 11.8; *P* = 0.02).

## Discussion

This RCT is the first in Australia to evaluate an intervention involving home visits by generalist CNs to optimise support for outpatients receiving SACT. The non-significant relative reduction in unplanned presentations of 12% was less than expected, with 31% of intervention group patients and 35% in the control group making one or more unplanned presentation across the first 3 systemic chemotherapy cycles.

Febrile neutropenia was the second most common reason for unplanned presentation in both groups. Febrile neutropenia carries a risk of infection, and the recommendation is that patients be urgently evaluated by a doctor [29] A Western Australian study reported that fever/febrile neutropenia, pain, nausea, vomiting and diarrhoea were the predominant reasons for unplanned admissions [30]. In our study, fever, febrile neutropenia, and nausea and vomiting were the three most common reasons for unplanned presentations in both groups. Where a CN made a provisional febrile neutropenia diagnosis based on clinical signs at assessment, the nurses followed the appropriate pathway and advised them to present to hospital or the relevant cancer centre. The intervention would not have prevented these unplanned presentations.

Another possible reason for the lack of difference in presentations between control and intervention groups is the high proportion of late-stage cancer patients recruited. Individuals with advanced cancer are more likely to experience a range of symptoms related to the disease rather than side effects of treatment that may lead to unplanned presentations. They have been reported as having a higher risk (subhazard ratio 1.92, 95% CI, 1.57–1.97) of making an emergency department visit [26].

In both groups, most participants reported comorbidities, particularly hypertension, heart disease and diabetes, as found in a large cancer cohort [31]. Cancer patients with such comorbidities have been reported as having a higher risk of emergency presentation [26].

In our study, results for the PROs were mixed. The CBI-B includes some of the physical, mental and social coping tasks faced by cancer patients and has been reported to be a measure of self-efficacy [17, 32]. Higher levels of self-efficacy in cancer patients have been associated with increased self-care and decreased physical and psychological symptoms [33, 34]. The intervention group had a statistically significantly higher CBI-B score than the control group at the start of cycle 4, indicating greater self-efficacy in coping with treatment side effects. While this increased self-efficacy did not result in a reduction in unplanned presentations, probably due to the serious nature of the predominant reasons for unplanned presentations, it is likely to have assisted patients to cope with the challenges that arose as they progressed through their cancer treatment, as reported in a qualitative component of this study [35].

A minimal clinically important difference (MCID) of 1.5 points [36] has been reported for the HADS anxiety and depression subscales. By the start of cycle 4, anxiety reduced for both groups, although only the intervention group showed a clinically significant reduction. Changes in depression for both groups did not reach the MCID.

HADS anxiety and depression scores ≥ 8 have been reported as cut-offs for indication of symptoms [37], and total scores ≥ 15 can indicate patients with a need for psycho-oncological care [38]. In our study, patients in both groups on average scored within the ‘normal’ range, and there may have been little opportunity for statistically significant between-group differences to be demonstrated.

While not directly related to the intervention, the finding that those with an informal carer had increased anxiety (HADS) and reduced self-efficacy (CBI-B) may be of interest to those researching the role carers play in the psychosocial outcomes of their family/friend with cancer, where findings have been mixed. Some studies report positive benefits [22–25], while other authors report social support can be perceived negatively by those impacted by a cancer diagnosis [39–42].

Despite the QLQ-C30 summary score reported to be as discriminative as the best-performing single QLQ-C30 for patients with solid tumours [43] and more sensitive to change than the subscales in patients with haematological malignancies [44], we found the intervention did not improve cancer-specific quality of life and symptoms as measured by the summary score or the 15 subscales. The relatively short timeframe for patients to complete three cycles (median 63 days), coupled with the majority of patients with stage IV cancer may have been a contributing factor. Research related to QoL has been mixed. An RCT of advanced cancer patients [45] did not find a difference in QLQ-C30 scores after 8 weeks and another RCT did not find intervention effects for QoL of advanced cancer patients using shorter timeframes, but did report on other studies that had found QoL benefits for interventions conducted for 12 weeks or more [46].

## Implications for practice

In this study, as most unplanned presentations were made during cycle 1 and during the first 7 days of a cycle a CN intervention targeting these could be considered. Socially isolated patients and those with comorbidities that complicated self-management of treatment side effects could also be considered [35]. Patients from culturally and linguistically diverse backgrounds would be of interest to study further, as well as development of an approach to identify which aspects make a patient at greater risk of presentation.

To address that very issue, there is currently a submission for funding, which draws on some of the ESCAPI findings, such as the at-risk timepoints and that trained CNs can improve cancer patients’ confidence in managing their symptoms, to deliver the model of care at each cycle of systemic treatment in the RPA (Royal Prince Alfred) Virtual Hospital. This is an initiative to provide 24/7 services through a virtual platform, connecting patients with skilled generalist nurses and multidisciplinary teams. The use of a patient-facing app to self-report symptoms will alert the virtual care nurse to provide support and/or care coordination.

## Strengths of the study

The intervention was delivered in the context of community health, which often experiences high staff turnover and fluctuating caseloads, such as during flu season. Throughout these challenges, the CNs were able to maintain the delivery of the service. The CN intervention increased patients’ self-confidence in managing symptoms and treatment side effects, highlighting the importance of including PROs in cancer trials, as the hospital utilisation data does not provide a complete picture of outcomes.

## Limitations of the study

The study was conducted in metropolitan Sydney only. Patients in the study were predominantly diagnosed as experiencing stage IV cancer. Cancer treatments changed over the period of the RCT study, including the introduction of immunotherapy and targeted therapies. Patients had to understand written or spoken English. As all cancer patients are advised to contact their GP if they feel the need, in this study, it was not possible to know to what extent the control group consulted their GP for advice and support and thereby affected the outcomes. The type of informal carer support and its frequency was not measured.

## Conclusion

In this study, a CN intervention did not reduce the number of unplanned presentations and hospital admissions. The study identified when presentations are most likely to occur, and for which symptoms. The intervention did improve patient confidence in managing cancer symptoms and side effects.

### Supplementary Information

Below is the link to the electronic supplementary material.

Supplementary file1 (DOCX 244 KB)
