# Correction to: Evaluating a shared care pathway intervention for people receiving chemotherapy to reduce post‑treatment unplanned hospital presentations: a randomised controlled trial

**DOI:** 10.1007/s00520-024-08326-4

**Published:** 2024-01-17

**Authors:** Judith Fethney, Bora Kim, Chantale Boustany, Heather McKenzie, Lillian Hayes, Keith Cox, Judy M. Simpson, Lisa G. Horvath, Janette L. Vardy, Jodi McLeod, Simon Willcock, Natalie Cook, Louise Acret, Kate White

**Affiliations:** 1 Susan Wakil School of Nursing and Midwifery, The University of Sydney, Sydney, Australia; 2 Cancer Care Research Unit, Sydney Local Health District, The University of Sydney, Sydney, Australia; 3 The Daffodil Centre, The University of Sydney, a joint venture with Cancer Council NSW, Sydney, Australia; 4 Chris O’Brien Lifehouse, Sydney, Australia; 5 Sydney School of Public Health, The University of Sydney, Sydney, Australia; 6 Faculty of Medicine and Health, The University of Sydney, Sydney, Australia; 7 Sydney Local Health District, Sydney, Australia; 8 Sydney District Nursing, Sydney Local Health District, Sydney, Australia; 9 MQ Health, Macquarie University Hospital, Primary Care, Sydney, Australia; 10 Healthdirect, Sydney, Australia

**Correction to: Supportive Care in Cancer (2023)**

https://doi.org/10.1007/s00520-023-08261-w

The error is as follows in the very last entry for Table 2:

**Table 2:** Cycle delivery, completion and delays of participants by treatment group

	Intervention N = 170 n (%)	Control N = 176 n (%)
Cycles commenced		
Cycle 1 only	4 (2.4)	7 (4.0)
Cycle 1 & 2	11 (6.5)	8 (4.5)
Cycle 1, 2 & 3	10 (5.9)	10 (5.7)
Cycle 1, 2, 3 & 4	145 (85.3)	151 (85.8)
Delay to treatment >7 days		
No	144 (84.7)	155 (88.1)
Yes	26 (15.3)	21 (11.9)
Deceased	6 (3.5)	4 (2.3)
Mean (SD) time at risk (days)	57.3 (16.4)	57.9 (16.2)
Range	8 to 112	3 to 97
Median (IQR)	63 (42–63)	63 (47–64)

Median (IQR) for the Control group is presented as 6 (47-64) in original article but should read 63 (47-64).

The original article has been corrected.

The publisher regrets this error.

