# Supplementary material for: Evaluating a shared care pathway intervention for people receiving chemotherapy to reduce post-treatment unplanned hospital presentations: a randomised controlled trial
Source: Support Care Cancer. 2024 Jan 3;32(1):77. doi: 10.1007/s00520-023-08261-w (PMC10764538; doi:10.1007/s00520-023-08261-w)
Supplement: Supplementary file 1 — Supplementary file1 (DOCX 244 KB) [file 520_2023_8261_MOESM1_ESM.docx]

**Resource 1**

**Information relating to PROs and sample size calculation for secondary outcomes**

The HADS is a valid measure for anxiety and depression in hospital and community settings [1]. Studies based on a range of cancer tumour types have reported Cronbach alpha coefficients of 0.80 – 0.89 for the Anxiety scale and 0.77 – 0.82 for the Depression scale [2, 3]. Anchor-derived criteria have been used to establish the minimal clinically important difference (MID) of 1.5 points for the HADS (both scales) [4] and 1.7 (both scales) [5]. With a final sample size of 326, the power to detect the minimal clinically important difference was estimated to be 92% for the HADS [6].

The CBI-B is a valid and reliable measure of self-efficacy, confidence and quality of life for people coping with cancer. A number of versions of the CBI exist: the 14 item CBI-B was derived from the CBI-L with 33 items, with the 14 item also further modified to a 12 item scale with total scale alpha coefficients ranging from 0.84 – 0.88 [7, 8, 9]. MIDs or effect sizes were not available at the time of development of the trial. A meta-analysis of 3,120 cancer patients across 37 controlled studies that used psychosocial measures to assess a range of cancer interventions reported an average effect size of 0.31 (95%CI: -0.13–0.75) [10], which was used in this study, similar to the effect size of 0.27 reported in a meta-analysis of interventions to enhance self-efficacy in cancer patients [11] and to the effect size of 0.28 proposed for the 14 item CBI-B by Giesler et al (2017) [12]. The sample size of 326 had 80% power to detect an effect size of 0.31 for the CBI-B [13] (G*Power 3.1.9.7 (Faul et al, 2009)).

The EORTC QLQ-C30 is an internationally used cancer specific questionnaire measuring physical, role, emotional, cognitive and social function as well as cancer symptoms and global quality of life. Reliability coefficients of 0.62- 0.92 have been reported [14]. MID for the EORTC QLQ-C30 has been reported as 10 points or more on the function and symptom scales [15, 16]. Using a minimum of 10 points and median standard deviations provided in [16], power ranged from 63.6 to 99.0, with an average of 92% [6] (StudySize 3.0 (Olofsson, 2001-2013)).

**Resource 2**

In the intervention group, there were six deceased patients, 19 who were withdrawn, and 25 patients who completed the trial but did not receive all six scheduled visits, resulting in an estimated 120/170 (71%) adherence rate to the full intervention.  There were seven patients whose CN activity data could not be accessed, and therefore this proportion may be slightly less. Of the 25 patients and the six scheduled visits, 15 missed one visit, four missed two visits, three missed three visits, two missed four visits and one patient missed all six scheduled visits. Reasons included making an unplanned presentation on the day of the visit, being admitted to hospital on day/days of visit, patient cancelling the visit, other activities planned on the day of the visit, administrative/communication errors as well as a number of visits that did not have the reason recorded. Missed CNs visits were evenly distributed across the three cycles.

**Resource 3**

Additional information relating to delays in treatment.

In the intervention group 26 patients (15%) had delays >7 days: 12 due to treatment toxicity or disease progression, 1 due to hospital admission from an unplanned presentation, 9 due to planned surgery and 4 due to social reasons. In the control group, 21 patients (12%) had delays >7 days: 12 due to toxicity or disease progression, 5 due to hospital admission from an unplanned presentation, 4 due to planned surgery or other planned procedure, and none due to social reasons. Planned surgery included procedures such as hysterectomy, debulking (removal of as much of a tumour as possible) and laparotomy. Social reasons included attending family functions and caring duties.

**Resource 4 EORTC analysis**

Analysis shows no significant difference on any of the 15 scales.


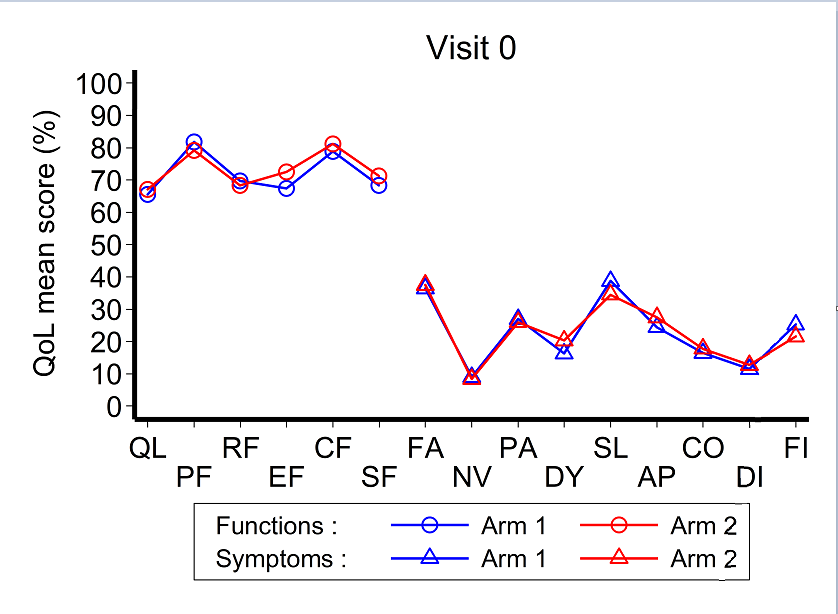

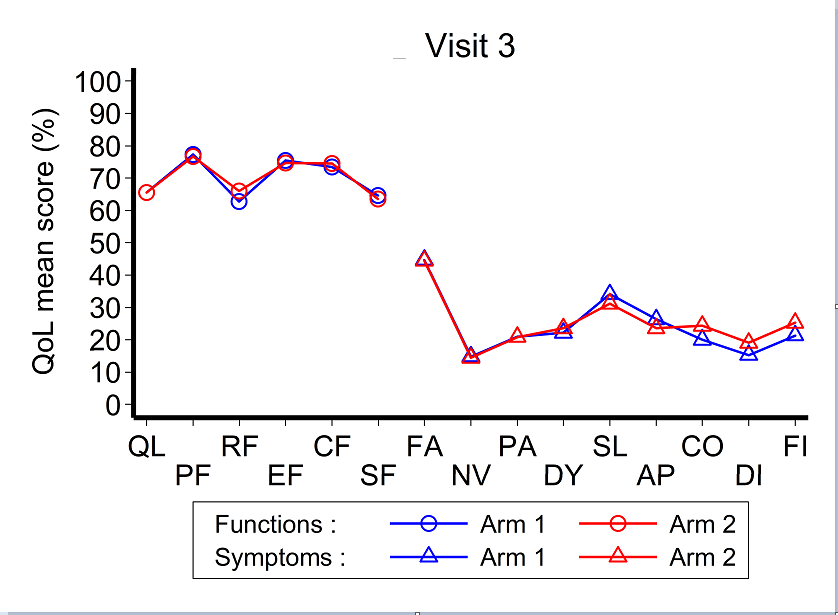


**Arm 1 = Intervention; Arm 2 = Control**

QL: Global health status (QoL) – 2 items DY: Dyspnea – 1 item

PF: Physical Functioning – 5 items SL: Insomnia – 1 item

RF: Role Functioning – 2 items AP: Appetite loss – 1 item

EF: Emotional Functioning – 4 items CO: Constipation – 1 item

C F: Cognitive functioning – 2 items DI: Diarrhea – 1 item

SF: Social functioning – 2 items FI: Financial difficulties – 1 item

FA: Fatigue – 3 items

NV: Nausea and vomiting – 2 items

PA: Pain – 2 items

These are the scales of greatest interest


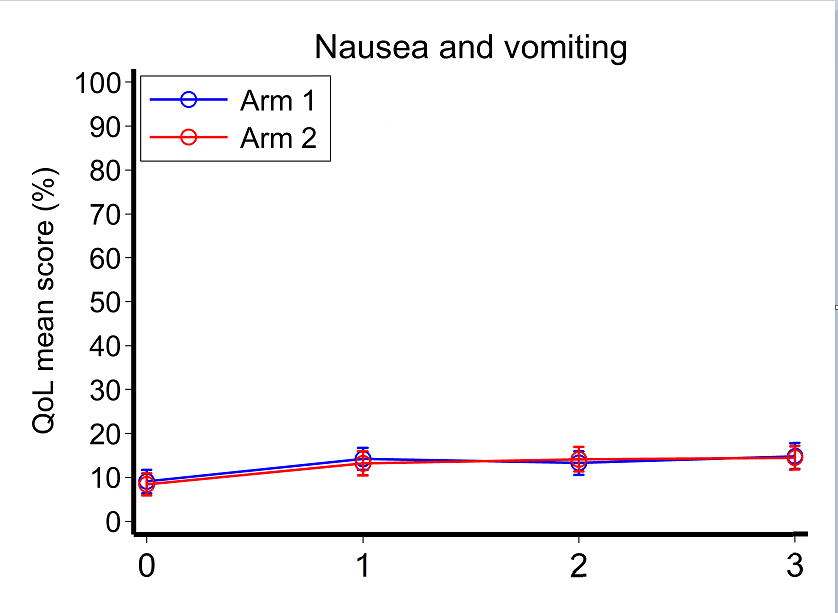

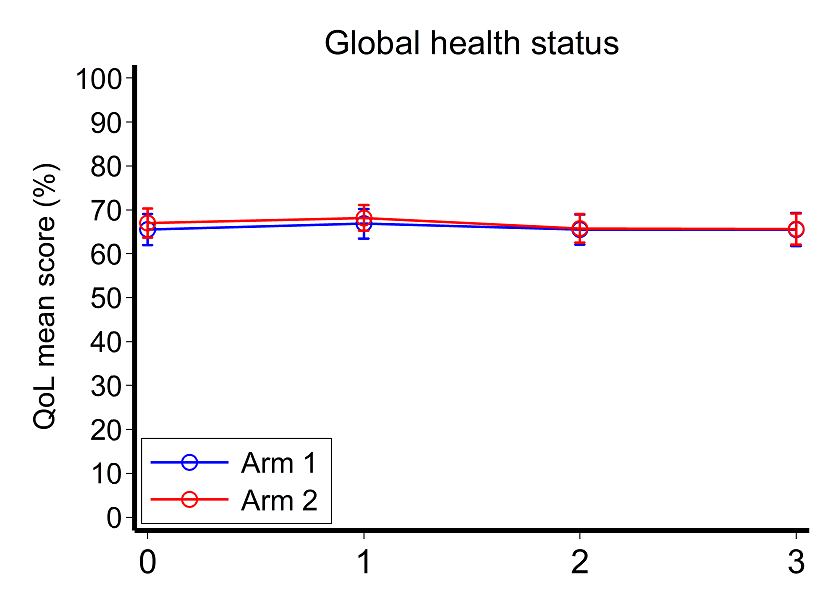


**Arm 1 = Intervention; Arm 2 = Control**

Australian reference values can be found at:

https://www.mja.com.au/journal/2019/210/11/eortc-quality-life-questionnaire-cancer-patients-qlq-c30-australian-general

**References for online resources**

1. Snaith, R.P. (2003) The Hospital Anxiety And Depression Scale. Health Qual Life Outcomes 1: 29. <https://doi.org/10.1186/1477-7525-1-29>

2. Zittoun R, Achard S, Ruszniewski M. (1999) Assessment of quality of life during intensive chemotherapy or bone marrow transplantation. Psychooncology 8(1): 64-73.

3. Lloyd-Williams M, Friedman T, Rudd, N. (2001) An Analysis of the Validity of the Hospital Anxiety and Depression Scale as a Screening Tool in Patients with Advanced Metastatic Cancer. J Pain Symptom Manage 22(6):990-996. doi:[10.1016/s0885-3924(01)00358-x](https://doi.org/10.1016/s0885-3924(01)00358-x)

4. Puhan, M., et al. (2008) The minimal important difference of the hospital anxiety and depression scale in patients with chronic obstructive pulmonary disease. Health Qual Life Outcomes, 6(1): 46. <https://doi.org/10.1186/1477-7525-6-46>

5. Lemay KR, Tulloch HE, Pipe AL, Reed JL(2019) Establishing the Minimal Clinically Important Difference for the Hospital Anxiety and Depression Scale in Patients With Cardiovascular Disease. J Cardiopulm Rehabil Prev. <http://doi.org/10.1097/HCR.0000000000000379>

6. StudySize 3.0, CREOSTAT HB, Sweden. <https://www.studysize.com/>

7. Heitzmann et al. (2011) Assessing self-efficacy for coping with cancer: development and psychometric analysis of the brief version of the Cancer Behaviour Inventory (CBI-B) Psychooncology 20(3):302-312. <https://doi.org/10.1002/pon.1735>

8. Chirico et al (2017) A meta-analytic review of the relationship of cancer coping self-efficacy with distress and quality of life. Oncotarget 8(22):pp 36800-36811. <https://doi.org/10.18632/oncotarget.15758>

9. Pereira M, Izdebski P, Pereira MG (2021) Validation of the Brief Version of the Cancer Behavior Inventory in Breast Cancer Portuguese Patients. J Clin Psychol Med Settings 28:491-502. <https://doi.org/10.1007/s10880-021-09773-5>

10. Rehse, B. and Pukrop, R. (2003) Effects of psychosocial interventions on quality of life in adult cancer patients: meta analysis of 37 published controlled outcome studies. Patient Educ Couns 50(2):179-186. https://doi.org/ [10.1016/s0738-3991(02)00149-0](https://doi.org/10.1016/s0738-3991(02)00149-0)

11. Merluzzi TV et al (2019) Interventions to enhance self‐efficacy in cancer patients: A meta‐analysis of randomized controlled trials. Psycho‐Oncology 28(9):1781-1790. <https://doi.org/10.1002/pon.5148>

12. Giesler JM et al (2017) Effect of a Website That Presents Patients’ Experiences on Self-Efficacy and Patient Competence of Colorectal Cancer Patients:Web-Based Randomized Controlled Trial J Med Internet Res 19(10). <https://doi.org/10.2196/jmir.7639>

13. Faul F, Erdfelder E, Lang AG, Buchner A (2007). G*Power 3: A flexible statistical power analysis for the social, behavioral, and biomedical sciences. Behavior Research Methods, 39, 175-191. https://www.psychologie.hh u.de/arbeitsgruppen/allgemeine-psychologie-und-arbeitspsychologie/gpower

14. King et al. (2007). Validity, reliability and responsiveness of the EORTC QLQ-C30 and the EORTC QLQ-LC13 in Australians with early stage non-small cell lung cancer, CHERE Working Paper 2007/13. CHERE, University of Technology, Sydney, Working Papers.

15. Osoba D et al (1998) Interpreting the significance of changes in health-related quality-of- life scores. J Clinical Oncol 16(1): 139-144. https://doi.org/ [10.1200/JCO.1998.16.1.139](https://doi.org/10.1200/jco.1998.16.1.139)

16. King M (1996) The interpretation of scores from the EORTC quality of life questionnaire QLQ-C30. Qual Life Res 5:555-567. <https://doi.org/10.1007/BF00439229>
